# Odontogenic orbital inflammation

**Published:** 2020

**Authors:** Victor Vlad Costan, Camelia Margareta Bogdănici, Liliana Gheorghe, Otilia Obadă, Cristian Budacu, Constantin Grigoraș, Daniela Gabriela Andronic, Irina Andreea Niagu

**Affiliations:** * Discipline of Oro-Maxillo-Facial Surgery, “Grigore T. Popa” University of Medicine and Pharmacy, Iași, Romania; “Sf. Spiridon” Hospital, Iaşi, Romania; ** Discipline of Ophthalmology, “Grigore T. Popa” University of Medicine and Pharmacy, Iași, Romania; “Sf. Spiridon” Hospital, Iaşi, Romania; *** Discipline of Radiology, “Grigore T. Popa” University of Medicine and Pharmacy, Iași, Romania; “Sf. Spiridon” Hospital, Iaşi, Romania

**Keywords:** orbital cellulitis, odontogenic, orbital decompression

## Abstract

**Objective:** This study aimed to determine the most frequent clinical aspects in patients with odontogenic orbital inflammation, the computed tomography (CT) aspect, and the most appropriate treatment.

**Material and Methods:** This is a retrospective case-series study conducted on 3 patients with ages between 16 and 55 years old, in the Ophthalmology and Oro-Maxillo-Facial Clinics of “Sf. Spiridon” Emergency Hospital, Iași, Romania. The following investigations were performed in all selected cases: visual acuity (VA), ocular motility examination, anterior segment examination at slit-lamp, fundus examination, intraoral clinical examination, sinus and orbital involvement on CT scan, pathogens involved.

**Results:** All three patients presented swelling of the genic and periorbital regions, conjunctival chemosis, hyperemia of the conjunctiva, proptosis, pain, decreased vision and extraocular movement restriction. The CT examination identified orbital and periorbital cellulitis and ethmoidal expanded maxillary sinusitis or pansinusitis. Dental extraction, transalveolar drainage and orbital decompression were performed in all three cases. The evolution was favorable with remission of proptosis, edema of the genic and periorbital regions and conjunctival chemosis. Visual acuity remained poor in one case due to total optic nerve atrophy.

**Conclusions:** Our study had a small number of patients, but the data was pertinent to ophthalmologists and maxillofacial surgeons who need to be aware of typical clinical features and the most common etiologies. Late treatment of dental infections can lead to severe ocular manifestations such as orbital cellulitis. Odontogenic orbital inflammation management involves a long-term and multidisciplinary approach.

**Abbreviations:** CT = computed tomography, VA = visual acuity, CBCT = cone beam computed tomography, TED = thyroid eye disease, MRI = magnetic resonance imaging, OOC = odontogenic orbital cellulitis, RAPD = relative afferent pupillary defect

## Introduction

The most frequent causes of orbital inflammation are the following: thyroid eye disease (TED), Wegener granulomatosis, sarcoidosis, histiocytic disorders, and xanthogranuloma. Orbital inflammation produces acute damage by leakage of proinflammatory cytokines, cells and intravascular fluid, into the extracellular space [**1**]. 

Orbital cellulitis represents one of the pathologies that can cause tension in the orbit. Orbital cellulitis can be caused by sinusitis, eyelid inflammation, dacryocystitis or hematogenous spread of infection [**2**]. Orbital inflammation is secondary to a variety of microorganisms such as *Mycobacterium tuberculosis, Candida albicans, Aspergillus*. Moreover, orbital involvement can be associated with neoplastic disorders such as *Human Herpes Virus 8* in Kaposi Sarcoma, *Epstein-Barr virus* in Hodgkin Lymphoma and *Human Papillomavirus 16 and 18* in squamous cell carcinoma [**3**]. Most cases of orbital cellulitis have a favorable prognosis, signs and symptoms remitting under antibiotic treatment or surgical drainage, without any complications. However, in some cases complications may occur such as extraocular muscle palsy, high intraocular pressure, pupil mydriasis or visual loss due to optic nerve damage [**2**]. 

Dental infections or interventions that have an important risk of spreading through paranasal sinuses to the orbital cavity are very rare and are associated with low prognosis [**4**]. 

Odontogenic orbital inflammation is a rare and severe condition, comprising only 2%-5% of all orbital cellulitis cases, and is associated with high risk of vision loss [**5**]. The inflammation spreads to the orbit infiltrating the apex of the dental roots, infecting the maxillary sinus and finally reaching the lower orbit infiltrating the inferior orbital fissure or through a defect of the orbital floor. The mechanisms of vision loss involve impairment of the anterior segment of the eye (exposure keratopathy), of the optic nerve (ischemic optic neuropathy), or of the retina (exudative retinal detachment). Odontogenic orbital inflammation involves a multidisciplinary and imagistic approach, antibiotic therapy and surgical treatment [**6**]. 

## Material and methods

This is a retrospective case-series study, conducted between December 2018 and January 2020, on 3 patients aged between 16 and 55 years, in the Ophthalmology and Oro-Maxillo-Facial Clinics of “Sf. Spiridon” Emergency Hospital, Iași, Romania. The following investigations were made in all selected cases: visual acuity (VA), ocular motility examination, anterior segment examination at slit-lamp, fundus examination, intraoral clinical examination, sinus and orbital involvement on computed tomography (CT) imaging, pathogens involved. Dental extraction and transalveolar drainage were performed in all the cases.

## Results

**Fig. 1:**
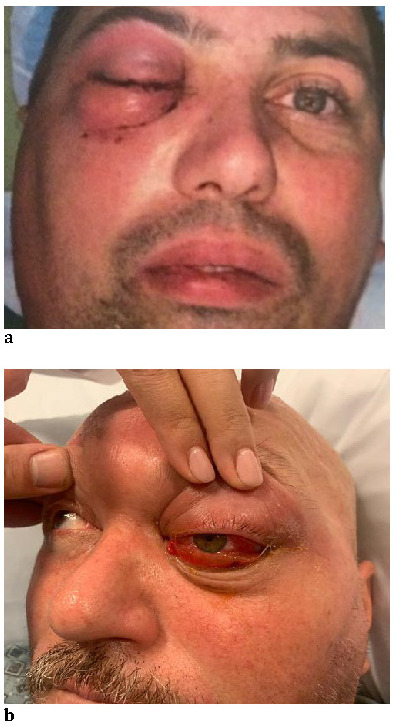
Initial appearance upon admission – (a) Case 1, (b) Case 2y

All three patients presented swelling of the genic and periorbital regions, conjunctival chemosis, hyperemia of the conjunctiva, proptosis, pain, decreased vision and extraocular movement restriction, with normal pupillary reactions and no detectable afferent pupillary defect (**Fig. 1**). There was no audible bruit on auscultation in any of the eyes. Fundus evaluation after pupillary dilatation with Tropicamide and Phenylephrine eye drops revealed pale borders of the optic disk (**Fig. 5a**). Patients did not have any history of ocular trauma. 

**Fig. 2:**
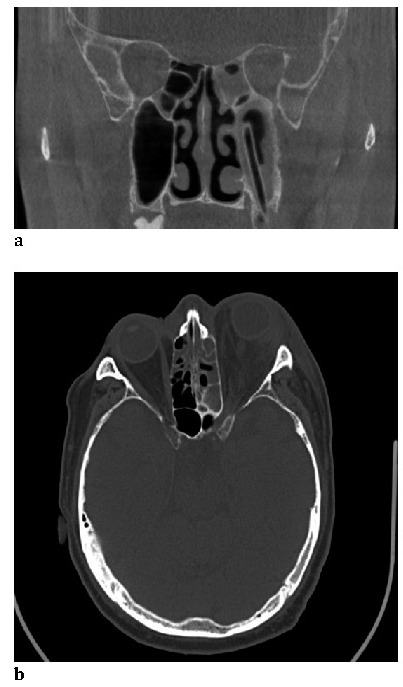
Sinus and orbital involvement on CT

All the patients had an initial investigation by computed tomography that confirmed the diagnosis – orbital and periorbital cellulitis and ethmoidal expanded maxillary sinusitis or in other cases pansinusitis (**Fig. 2a, b**). Dental radiography diagnosed a maxillary abscess unraveling the odontogenic origin. 

They were admitted in the Oro-Maxillo-Facial Clinic or in the Ophthalmology Clinic, where the antibiotic treatment was started – Ceftriaxone, Clindamycin or Vancomycin, associated with Dexamethasone. The patients were evaluated daily by both specialists.

In one case, the culture from the external orbital angle indicated *Streptococcus constellatus*, (a bacteria from *Peptostreptococcus family*), a saprophyte originated in the oral mucosa, but in other cases, the microbiological examination for aerobic bacteria revealed the absence of growth at 48 hours.

**Fig. 3:**
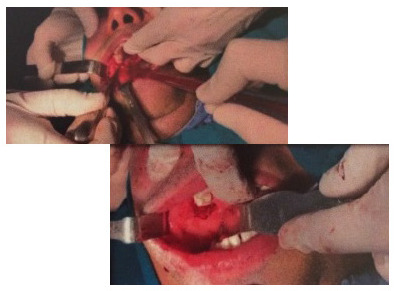
Dental extraction and transalveolar drainage – Case 1

**Fig. 4:**
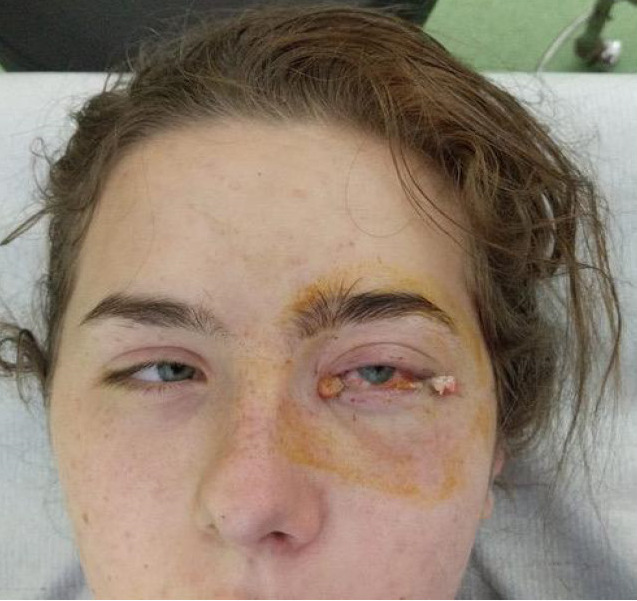
Orbital decompression – Case 3

**Fig. 5:**
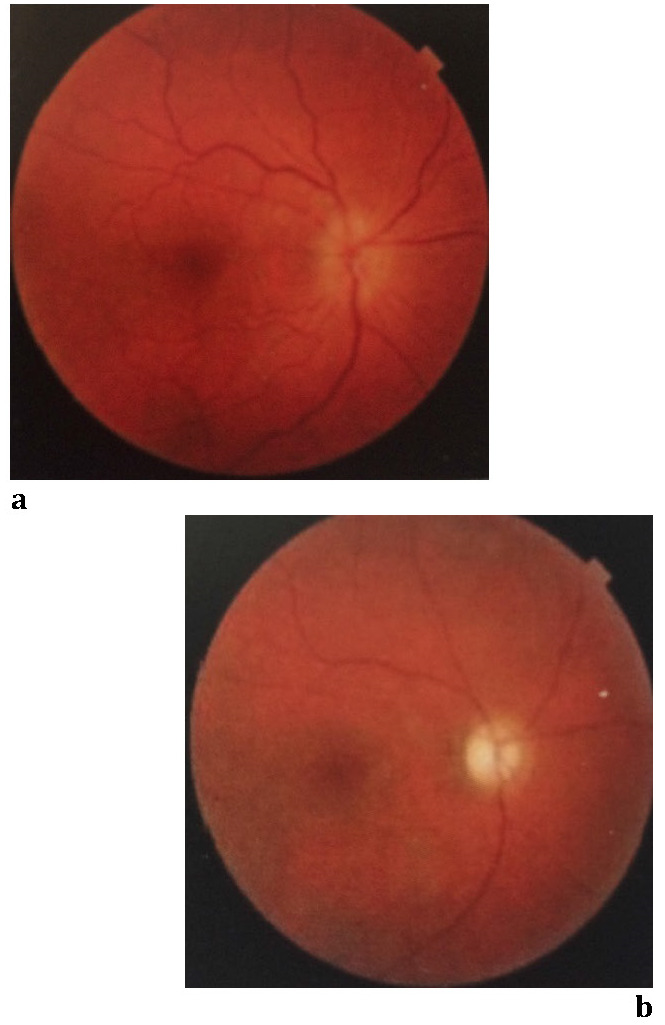
RE: Fundus appearance on admission (a) and on last examination (b) – Case 1

**Fig. 6:**
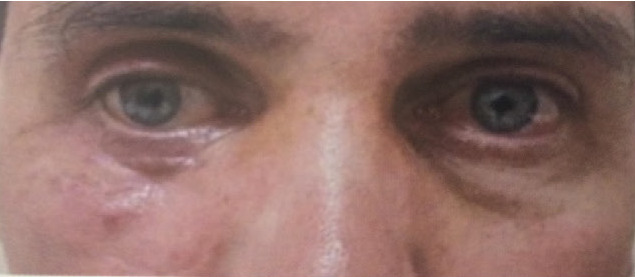
RE: Appearance of the periocular region on last examination - Case 1

Dental extraction, transalveolar drainage (**Fig. 3**) and orbital decompression (**Fig. 4**) were performed in all three cases. In one case, the patient also had retrobulbar injection with Dexamethasone and Vancomycin. Post-operative, the patients started to show improvement in ocular movements and reduced proptosis. Visual acuity remained poor in one case due to total optic nerve atrophy (**Fig. 5b**). All the patients were kept under observation with monthly follow-up up to six months to rule out local recurrence. The evolution was favorable with remission of proptosis, edema of the genic and periorbital regions and conjunctival chemosis (**Fig. 6**).

Three weeks after the surgical drainage and antibiotic treatment, the acute maxillary sinusitis became chronic in all the cases. The chronic maxillary sinusitis was cured at the same time with the closing of the oro-antral communication (due to the transalveolar drainage) through a Caldwell-Luc approach.

## Discussions

The most frequent symptoms of orbital affection are represented by swelling of eyelids and conjunctiva, hyperemia, epiphora, discomfort or displacement of the eye. 

Bacterial orbital inflammation represents a critical infection affecting the soft tissues at the back of the orbital septum making it life-threatening. It does not depend on age but it is more frequent in children. The more frequent pathogens are *Streptococcus pneumoniae, Staphylococcus aureus, Streptococcus pyogenes and Haemophilus influenzae*. Typically, the origin of the infection is from the paranasal sinuses [**7**]. Fungal infections are always a possibility, especially in immunodepressed patients. The most common source of orbital cellulitis is bacterial rhinosinusitis. Approximately 86-98% of the cases with orbital cellulitis have coexisting rhinosinusitis [**8**]. Origin of the infection can also be preseptal cellulitis, dacryocystitis, midfacial skin or dental infection and posttraumatic, even any type of ocular surgery. The condition usually develops with fever and peripheral leukocytosis and the diagnosis is indicated by CT scan or MRI [**7**,**8**]. 

Odontogenic orbital cellulitis (OOC) is a rare and severe cause of orbital infection, with a bad evolution [**9**]. 

In a prior series, 45.8% of the patients with OOC had a final vision of light perception or worse. In our study, only one patient had a visual acuity uncertain light perception, the others had a best corrected VA > 0.1. It is important to always evaluate for potential complications such as the development of intracranial extensions, destructive sinus disease, orbital abscess or vision loss [**10**], as the case of our patient who presented optic disc edema on admission and permanent vision loss due to secondary optic nerve atrophy.

The OOC infection spreads most frequently through the paranasal sinuses, and less frequently through the premaxillary soft tissues reaching the orbit [**11**], all our patients having a confirmed diagnosis of sinusitis. All the patients had no history of trauma or skin infection, but all had maxillary sinusitis or pansinusitis. 

Odontogenic orbital infection of the orbit is a rare condition. Apical osteitis or infected tooth sockets after surgical teeth removal or trauma can be the reason of odontogenic infection. The infection spreads through the maxillary sinus, the canine fossa with a thrombophlebitis of the angular vein or the pterygopalatine fossa and infra-temporal fossa both reaching the orbit by means of the inferior orbital fissure [**12**]. 

Clinically, orbital cellulitis is dominated by swelling and pain. Patients with odontogenic orbital inflammation present with symptoms or history that are highly suggestive for this condition’s etiology [**13**]. This study identified the most common initial presentations such as proptosis, chemosis, vision loss, reduced eye movements. Even though orbital cellulitis is usually associated with diplopia and RAPD (relative afferent pupillary defect), this study did not reveal patients with any of these symptoms. Secondary to the incomplete growth of the paranasal sinuses and the thin bony barrier between the orbit and the anterior skull base present in young patients, the condition is more frequent in this age group [**14**]. 

Many reports in literature identified the bacteriologic aspect of the acute orbit. *Staphylococcus aureus, Streptococci pneumoniae, and Hemophilus influenza* are predominantly responsible for these infections. Anaerobic bacteria are present in chronic sinusitis and are rare in the acute orbit caused by sinusitis [**12**]. In our cases, one culture indicated *Streptococcus constellatus* and the microbiological examination for aerobic bacteria revealed the absence of growth at 48 hours for the other. 

Most cases with orbital cellulitis have a favorable prognosis and can be treated with oral or intravenous antibiotics. In our study, all the cases were managed with antibiotics such as Ceftriaxone, Clindamycin or Vancomycin. Dental extraction and transalveolar drainage were performed for all patients, with a favorable evolution. 

Management of odontogenic orbital inflammation in immunocompromised patients includes surgical procedures and medical therapy in a multidisciplinary approach [**15**]. 

Our study had a small number of patients, but the data was pertinent to the general ophthalmologists and maxillofacial surgeons who need to be aware of typical clinical features and the most common etiologies. 

## Conclusions

Late treatment of dental infections can lead to severe ocular manifestations such as orbital cellulitis. Orbital inflammation diagnosis is expensive and time-consuming needing complex investigations such as CT imaging or sinus cone beam computed tomography (CBCT). Odontogenic orbital inflammation management involves a long-term and multidisciplinary approach.

**Disclosures**

None.

**Conflicts of interest**

None.
